# Shifting from Proactive to Reactive Control: Cognitive Control in Action Video Game Players with Gaming Disorder

**DOI:** 10.3390/bs16061022

**Published:** 2026-06-18

**Authors:** Yuhong Zhou, Jiayu Li, Danni Zhan, Zijie Fang, Xuemei Gao

**Affiliations:** 1 Psychological Research and Counselling Center, Southwest Jiaotong University, Chengdu 611756, China; zhouyh@swjtu.edu.cn (Y.Z.); jasonleon@my.swjtu.edu.cn (Z.F.); 2 Department of Psychology, Hebei Normal University, Shijiazhuang 050024, China; lijiayu0104@hebtu.edu.cn; 3 Department of Vocational Education, The Open University of Mianyang, Mianyang 621000, China; 18184004946@163.com

**Keywords:** action video games, gaming disorder, cognitive control, dual mechanisms, task switching

## Abstract

While action video games (AVGs) can enhance cognitive control, mechanisms underlying gaming disorder (GD) remain unclear. Using the Dual Mechanisms of Control framework, two task-switching experiments dissociated proactive and reactive control among AVG players with GD, recreational game users (RGU), and non-gamers (NG). Experiment 1 provided initial evidence that, unlike healthy controls, GD players showed difficulty sustaining proactive preparation over extended intervals and tended to rely more on post-response interference resolution. Experiment 2 further supported this reactive dependence: after prolonged delays, switch costs in the GD group dropped to negligible levels, whereas residual costs persisted in RGU and NG groups. These findings provide converging evidence that GD players exhibit relatively fragile proactive control and a compensatory over-reliance on reactive control. Consequently, cognitive impairment in GD reflects a shift in processing mode rather than a generalized deficit, highlighting mechanism-specific targets for clinical interventions.

## 1. Introduction

Gaming is a popular leisure activity in the digital era, with the global gamer population estimated to reach 3.42 billion in 2024 (Newzoo, 2024). Action video games (AVGs) are particularly popular (Statista, 2026). These games often impose substantial demands on cognitive control, as players must respond flexibly to rapidly changing and unpredictable environments while shifting attention across multiple task-relevant targets (Dale et al., 2020). Based on these characteristics, previous research has shown that AVG experience is associated with improvements in cognitive control (Bediou et al., 2018; Zhou et al., 2026), a set of processes that enables individuals to guide thoughts and behavior in line with internally maintained goals (Braver, 2012). However, excessive involvement in gaming may progress to Gaming Disorder (GD), a condition marked by impaired control over gaming and continued engagement despite negative consequences, ultimately resulting in functional impairments (World Health Organization [WHO], 2022). Impaired cognitive control is recognized as one of the core symptoms of GD (Wang et al., 2021). This gives rise to a key question: if AVG experience is associated with cognitive advantages, how is cognitive control and its underlying mechanisms manifested in AVG players with GD?

The Dual Mechanisms of Control (DMC) framework may help explain this question. According to this framework, cognitive control operates in two distinct modes: proactive control and reactive control. Proactive control involves the sustained and anticipatory maintenance of goal-relevant information before interference occurs, reflecting top-down early selection, whereas reactive control is recruited only after interference emerges, serving as a bottom-up late correction mechanism (Braver, 2012). Cue informativeness is typically used to distinguish these two modes: informative cues are more likely to engage or index proactive control, whereas uninformative cues place greater reliance on reactive control (Braver, 2012; Czernochowski, 2014; MacDowell et al., 2022). In task-switching paradigms, switch costs are widely used behavioral indices of cognitive control, reflecting the additional cost of reconfiguring task sets and resolving interference from the previous task (Monsell, 2003). The DMC framework has been widely applied to characterize variations in cognitive control across different populations, including individuals with schizophrenia, ADHD, and depression, and gamers (Cai et al., 2023; Fathi et al., 2022; Vanderhasselt et al., 2014; Lesh et al., 2013).

To date, research investigating cognitive control within the DMC framework in the context of gaming remains sparse, particularly concerning GD. Existing evidence suggests that, compared with healthy controls, individuals with GD show impaired proactive control (Fathi et al., 2022), and that reductions are also associated with AVG experience (Bailey et al., 2010; Cudo et al., 2024). Notably, recent work suggests that AVG experiences and GD severity jointly shape the internal mechanisms of cognitive control. Specifically, in players with GD, richer AVG experiences are associated with enhanced reactive control and reduced proactive control, whereas in non-GD players, richer AVG experiences are associated with enhanced proactive control and reduced reactive control (Cudo & Kopiś-Posiej, 2026).

The above shift in control modes may be further understood through the broader perspective of dual process theory, specifically the reflective–impulsive model (Strack & Deutsch, 2004). According to this model, behavior is jointly shaped by a reflective system, which generates decisions based on knowledge and values and reflects top-down control, and an impulsive system, which is more strongly driven by bottom-up peripheral input and more immediate, stimulus-driven responding. The reflective system depends heavily on cognitive resources, whereas the impulsive system requires relatively little capacity and is therefore more likely to dominate behavior under suboptimal conditions (Strack & Deutsch, 2004). Consistent with this resource dependence, AVG players have been found to possess superior working memory and cognitive control capacity (Yao et al., 2020; Zhou et al., 2026), which likely facilitates the sustained maintenance of task goals characteristic of proactive control. Conversely, GD is often associated with a hyperactive impulsive system that overwhelms reflective processes (Şalvarlı & Griffiths, 2022; Zhou et al., 2022), thereby potentially shifting the cognitive balance from anticipatory preparation toward reactive, on-demand interference resolution. Identifying such divergent control profiles is essential for distinguishing pathological gaming from high engagement and for informing targeted cognitive remediation strategies.

### The Current Study

The present study comprised two sequential experiments designed to dissociate proactive and reactive control and to elucidate the unique cognitive mechanisms underlying AVG players with GD. Experiment 1 employs a cued task-switching paradigm, manipulating the Cue–Stimulus Interval (CSI) and Response–Cue Interval (RCI) to index proactive and reactive control, respectively. Building upon the initial findings, Experiment 2 utilizes an uncued design and extends the Response–Stimulus Interval (RSI) to 3000 ms to provide a finer temporal resolution of the reactive process. This combined design allows us to determine whether AVG players with GD can utilize anticipatory cues for active goal maintenance (i.e., proactive control) or whether they rely predominantly on the passive, time-dependent dissipation of task-set interference (i.e., reactive control). The hypotheses are as follows:

**H1.**  *Compared to AVG players with GD and non-gamers (NG), recreational game users (RGU) will exhibit superior proactive control. Specifically, RGU will show a significantly larger reduction in switch costs under long CSI conditions. This effect will be more pronounced under low-RCI conditions (Experiment 1).*

**H2.**  *AVG players with GD will demonstrate an increased reliance on reactive control relative to RGU and NG. Specifically, GD will show a significantly larger reduction in switch costs under long RCI (Experiment 1) or RSI (Experiment 2) conditions. This effect will be more pronounced under low-CSI conditions in Experiment 1.*

## 2. Experiment 1: Cued Task-Switching Task

Experiment 1 employed a cued task-switching paradigm to index proactive and reactive control via CSI and RCI, respectively. By comparing AVG players with GD, RGU, and NG, we tested whether the RGU group exhibited a proactive control advantage, and whether the GD group demonstrated a specific reliance on reactive control.

### 2.1. Materials and Methods

#### 2.1.1. Participants

A total of 887 participants were initially recruited through online advertisements and completed the initial screening. Based on the predefined inclusion criteria described below, 131 participants were eligible for the laboratory experiment, including 38 participants with GD, 39 Recreational Game Users (RGU), and 54 Non-Gamers (NG). After excluding participants who discontinued during practice due to task difficulty (*n* = 4; GD = 1, RGU = 1, NG = 2) or failed to meet the performance criterion (accuracy < 80%, *n* = 8; GD = 4, NG = 4), the final sample comprised 119 participants (aged 17–26 years; M = 20.01, SD = 1.56): GD group (*n* = 33), RGU group (*n* = 38), and NG group (*n* = 48). All participants were right-handed and reported no history of psychiatric disorders and brain injury. The study was approved by the Institutional Review Board of our university and conducted in accordance with the Declaration of Helsinki. All participants provided written informed consent and were compensated.

Based on established criteria for action video game (AVG) players (Green et al., 2017) and GD (Chen et al., 2020; Dong et al., 2018), participants were classified into three groups. In the present study, GD refers to AVG players meeting screening criteria for Gaming Disorder rather than clinically diagnosed patients. The GD group met the following conditions: (1) played shooter, action-RPG, sports, driving, adventure, or MOBA games for ≥14 h/week over the past year; (2) AVGs accounted for ≥50% of total gaming time in the past year; (3) had ≥ 1 year of AVG experience; and (4) had an IAT score ≥ 50 and met ≥ 5 DSM-5 criteria. The RGU group’s inclusion criteria were identical to those of the GD group, except they required an IAT score < 50 and met ≤ 4 DSM-5 criteria. Lastly, the NG group consisted of individuals who played AVGs ≤ 5 h/week, had a total gaming time ≤ 7 h/week, scored < 50 on the IAT, and met ≤ 4 DSM-5 criteria. Reported popular AVGs included PUBG Mobile, CrossFire, Overwatch, Honor of Kings, League of Legends, Assassin’s Creed, and QQ Speed.

Demographic and gaming characteristics of the final experimental sample are summarized in Table 1. The three groups did not differ significantly in gender distribution, age, or years of education (*p*s > 0.05). The GD group exhibited significantly higher weekly AVG hours, IAT, and DSM-5 scores compared to the RGU group, followed by the NG group (GD > RGU > NG, *p*s < 0.05). For BDI scores, the GD group scored significantly higher than both the RGU and NG groups (*p*s < 0.05), with no significant difference between the latter two (*p* > 0.05). Additionally, the GD and RGU groups reported significantly more weekly non-AVG hours than the NG group (*p*s < 0.05) but did not differ from each other (*p* > 0.05).

#### 2.1.2. Materials and Procedure

We employed a cued task-switching paradigm using bivalent, incongruent stimuli, programmed in E-Prime 1.1 (Psychology Software Tools, Inc., Pittsburgh, PA, USA) on a 17-inch Dell monitor (Dell Technologies Inc., Round Rock, TX, USA). The stimuli consisted of four Chinese color words (red, green, yellow, and blue), presented in 36-point bold Songti font in black and superimposed on colored squares (red, green, yellow, or blue; 1.76 cm × 1.76 cm). The task was designed to include only incongruent stimuli, such that the semantic meaning of the word never matched the color of the square. This allowed us to examine task-set interference under a consistent level of stimulus conflict while avoiding the introduction of stimulus congruency as an additional within-subject factor. This design yielded 12 unique incongruent stimulus combinations.

A spatial cue (an asterisk) presented above or below the stimulus indicated whether participants should perform the color-naming or word-naming task. The cue-to-task mapping was counterbalanced across participants. Responses were recorded using four keys on a standard keyboard (“D,” “F,” “J,” and “K”), corresponding to red, green, yellow, and blue, respectively. Participants were instructed to place their left and right index and middle fingers on these keys, and the color-to-key mapping was counterbalanced across participants.

**Figure 1:**
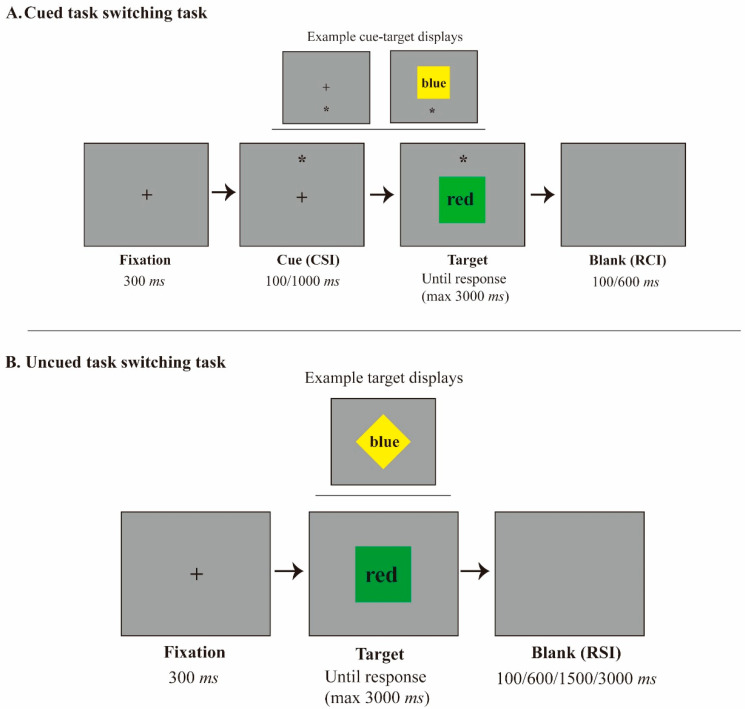
Task procedures for Experiments 1 and 2. (**A**) Cued task-switching task in Experiment 1, with CSI and RCI manipulated across blocks. Participants performed either the color-naming or word-naming task according to the spatial cue (e.g., upper asterisk = color-naming and lower asterisk = word-naming, or vice versa), with the cue-task mapping counterbalanced across participants. (**B**) Uncued task-switching task in Experiment 2, with RSI manipulated across blocks. Participants performed either the color-naming or word-naming task according to the background shape (e.g., diamond = color-naming and square = word-naming, or vice versa), with the shape-task mapping counterbalanced across participants.

The experiment followed a 3 (Group: GD, RGU, NG) × 2 (CSI: 100 ms vs. 1000 ms) × 2 (RCI: 100 ms vs. 600 ms) mixed design, with Group as a between-subjects factor and CSI and RCI as within-subjects factors. The four CSI × RCI combinations were administered across four separate blocks, with block order counterbalanced across participants. Each block contained 101 trials. After excluding the first trial of each block, which could not be classified as a switch or repeat trial, each CSI × RCI condition included 100 classified trials, with switch and repeat trials occurring in equal proportions. Trials were presented in a pseudorandom sequence: identical color squares or words were not presented on consecutive trials, and both the color and word meaning differed between adjacent trials. Participants were given a 2 min rest break between blocks. Before the formal task, participants completed a 52-trial practice phase and were required to achieve at least 80% accuracy before proceeding. The task procedure is illustrated in Figure 1A.

#### 2.1.3. Data Analysis

Data were analyzed using linear mixed models (LMMs), which are well suited for unbalanced repeated measures data while accounting for participant level random variation (Van der Elst et al., 2013). Because switch costs were computed from participant-level condition means rather than trial-level RTs, each participant contributed only four repeated observations per experiment. We therefore used random-intercept models to account for individual differences in overall switch costs, as adding random slopes would have overparameterized the models.

Descriptive statistics and preliminary group comparisons were conducted in JASP 0.19.1 (JASP Team, University of Amsterdam, Amsterdam, the Netherlands), and all LMM analyses were performed in R 4.4.1 (R Foundation for Statistical Computing, Vienna, Austria) using the lmerTest and emmeans packages. For the RT analysis, the first trial of each block was excluded because it could not be classified as repeat or switch (0.99% of total trials). Among the remaining trials, trials with incorrect responses (7.89%), RTs shorter than 200 ms (0.01%), or RTs exceeding ± 3 SD (0.48%) from the mean under each condition were excluded. Overall, this procedure excluded 9.36% of trials from the RT analysis. There were no significant group differences in either mean RT (GD: 1208.31 ± 195.18 ms, RGU: 1241.26 ± 152.56 ms, NG: 1248.56 ± 164.98 ms, *F*(2, 116) = 0.58, *p* = 0.56, $\eta_{p}^{2}$ = 0.01) or error proportions (GD: 7.92% ± 4.58%, RGU: 7.76% ± 3.56%, NG: 8.15% ± 3.76%, *F*(2, 116) = 0.11, *p* = 0.90, $\eta_{p}^{2}$ = 0.002) among the GD, RGU, and NG groups. RT switch cost served as the primary dependent variable, as it is a standard behavioral index of task switching performance (Monsell, 2003). It was defined as the mean of two task-specific switch costs: color switch cost (color-switch minus color-repeat trials) and word switch cost (word-switch minus word-repeat trials). The accuracy switch cost was further examined in supplementary analyses to assess whether the RT findings could be explained by the speed–accuracy trade-off.

Group (GD, RGU, NG) was treated as a between-subject factor, and participant was modeled with a random intercept (1|ID). The primary model was specified as SwitchCost ~ Group × CSI × RCI + (1|ID). Fixed effects were evaluated using Type III tests. Because the present study focused on group-related effects, significant interactions involving Group were followed by simple effects analyses using estimated marginal means, with Holm correction applied to multiple comparisons. To examine the robustness of group-related effects, a second model included weekly AVG hours and BDI score as grand-mean-centered covariates. Model diagnostics showed that all LMMs converged successfully without singular fits, with no serious deviations from residual normality or homoscedasticity.

### 2.2. Results

**Figure 2:**
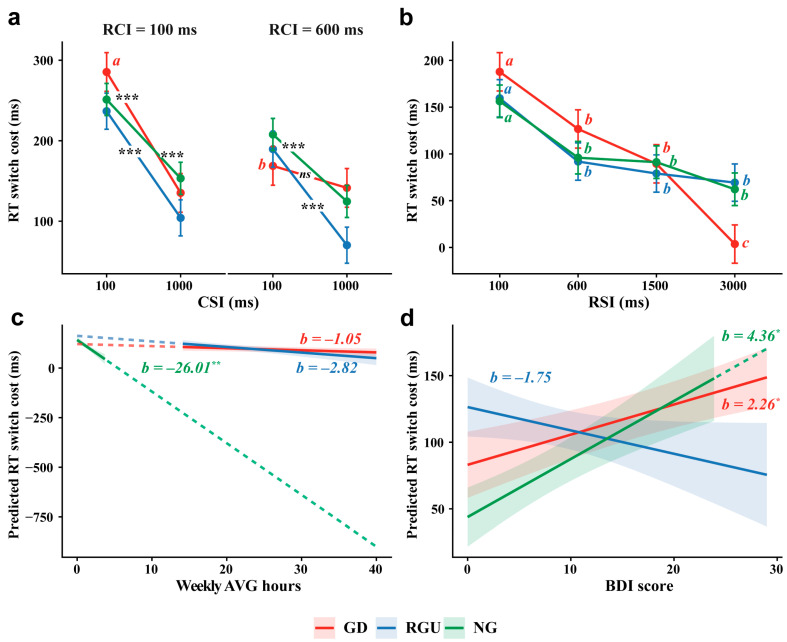
Simple effects and simple slope analyses. (**a**) Simple effects of the Group × CSI × RCI interaction. Asterisks indicate significant differences between the two CSI conditions within each RCI condition, whereas lowercase letters indicate differences between RCI conditions within each CSI condition. (**b**) Simple effects of the Group × RSI interaction. Lowercase letters indicate pairwise differences between adjacent RSI conditions within each group. (**c**) Group-specific simple slope plot of predicted switch cost as a function of weekly AVG hours. (**d**) Group-specific simple slope plot of predicted switch cost as a function of BDI score. In Panels (**a**,**b**), identical lowercase letters indicate no significant difference, whereas different lowercase letters indicate a significant difference; letter colors correspond to the respective groups. All simple-effect and pairwise comparison annotations in Panels (**a**,**b**) are based on Holm-corrected post hoc tests. In Panels (**c**,**d**), solid lines represent fitted values within the observed data range, whereas dashed lines indicate extrapolated model predictions beyond the observed range. Error bars and shaded bands represent ±1 SE. * *p* < 0.05, ** *p* < 0.01, *** *p* < 0.001, *ns* indicates nonsignificant.

The omnibus test of fixed effects in LMM revealed significant main effects for CSI (*F*(1, 357) = 100.13, *p* < 0.001, $\eta_{p}^{2}$ = 0.219) and RCI (*F*(1, 357) = 18.66, *p* < 0.00$1,\eta_{p}^{2}=0.05$); switch costs were significantly lower at CSI 1000 than CSI 100 (121.45 vs. 223.21 ms) and at RCI 600 than RCI 100 (150.36 vs. 194.29 ms). The CSI × RCI interaction was significant (*F*(1, 357) = 6.10, *p* < 0.05, $\eta_{p}^{2}$ = 0.017), and the Group × CSI × RCI interaction was marginally significant (*F*(2, 357) = 2.95, *p* = 0.054, $\eta_{p}^{2}$ = 0.016). Decomposition of the three-way interaction (Figure 2a) revealed distinct profiles across groups as follows:

Proactive Control (CSI effects). At the 100 ms RCI, all groups exhibited a significant CSI effect (*p*s < 0.001), with switch costs decreasing when preparation time increased to 1000 ms (∆SwitchCost: GD: 150.24 ms; RGU: 132.53 ms; NG: 97.92 ms). However, at the 600 ms RCI, this CSI effect persisted only for the RGU (∆SwitchCost: 119.38 ms, *p* < 0.001) and NG (∆SwitchCost: 83.24 ms, *p* < 0.001) groups, while it became non-significant for the GD group (∆SwitchCost: 27.28 ms, *p* = 0.32).

Reactive Control (RCI effects). At the 100 ms CSI, only the GD group exhibited a significant RCI effect, with switch costs significantly decreasing as RCI extended from 100 to 600 ms (∆SwitchCost: 116.67 ms, *p* < 0.001). Neither the RGU (∆SwitchCost: 47.09 ms, *p* = 0.065) nor the NG group (∆SwitchCost: 43.42 ms, *p* = 0.056) showed such a reliance on temporal dissipation. At the 1000 ms CSI, no significant RCI effects were observed in any group (*p*s > 0.05).

Robustness analyses, controlling for weekly AVG hours and BDI score, yielded consistent results (see Table 2), suggesting that these group-specific control patterns were unlikely to be driven by AVG gaming volume or depressive symptoms. In addition, supplementary ACC switch-cost analyses showed no pattern opposite to the RT results, arguing against a speed–accuracy trade-off explanation (see Appendix A). In summary, these results provide partial support for H2 and did not support H1, indicating a tendency toward greater reliance on reactive control in AVG players with GD but no categorical proactive superiority in the RGU group.

## 3. Experiment 2: Uncued Task-Switching Task

To verify the above reactive-dependent mechanism in GD, Experiment 2 employed an uncued paradigm. By extending the RSI up to 3000 ms, we sought to further confirm the unique reactive control pattern in the GD group.

### 3.1. Materials and Methods

#### 3.1.1. Participants

Experiment 2 was conducted in the same laboratory session and with the same invited experimental sample as Experiment 1. All participants completed Experiment 1 first, followed by a 10 min rest break, and then completed Experiment 2. Of the 131 eligible participants invited after the initial screening, three participants (GD = 1, RGU = 1, NG = 1) discontinued during practice due to task difficulty and an additional four (GD = 1, NG = 3) were excluded for failing to meet the performance criterion (overall accuracy < 80%), resulting in a final sample of 124 participants (GD: *n* = 36; RGU: *n* = 38; NG: *n* = 50). Demographic and gaming-related distributions remained consistent with the patterns detailed in Experiment 1 (see Table 1).

#### 3.1.2. Materials and Procedure

Experiment 2 employed an uncued task-switching paradigm (Yang, 2017) to isolate reactive control. The task was programmed in E-Prime 1.1 and presented on a 17-inch monitor, using bivalent, incongruent stimuli with the same color words, font, stimulus size, and response keys as in Experiment 1. The stimuli consisted of black Chinese color words superimposed on colored squares or diamonds; the diamond background was created by rotating the square background by 45°. Unlike Experiment 1, there was no explicit spatial cue; instead, the background shape served as the implicit task cue, and participants performed either the color-naming or word-naming task depending on the shape. The shape-to-task mapping, as well as the color-to-key mapping, was counterbalanced across participants.

The experiment followed a 3 (Group: GD, RGU, NG) × 4 (RSI: 100 ms, 600 ms, 1500 ms, 3000 ms) mixed design, with Group as a between-subjects factor and RSI as a within-subjects factor. The four RSI conditions were administered across four separate blocks, with block order counterbalanced across participants. Each block contained 101 trials and followed the same trial structure and pseudorandom constraints as Experiment 1; after excluding the first trial of each block, each RSI condition included 100 classified trials, with equal proportions of switch and repeat trials. Participants were given a 2 min rest break between blocks. Before the formal task, participants completed 40 practice trials and were required to achieve at least 80% accuracy before proceeding. The task procedure is illustrated in Figure 1B.

#### 3.1.3. Data Analysis

Data cleaning and switch costs calculations were identical to those in Experiment 1. Participants with an overall accuracy below 80% were excluded. For the RT analysis, the first trial of each block (0.99% of total trials), incorrect trials (7.08%), RTs shorter than 200 ms (0.004%) and RT outliers exceeding ±3 SD (0.68%) from the mean of each RSI condition were removed, resulting in the exclusion of 8.75% of total trials. There were no significant group differences in either mean RT (GD: 1216.51 ± 206.75 ms, RGU: 1259.28 ± 191.59 ms, NG: 1272.43 ± 196.75 ms, *F*(2, 121) = 0.87, *p* = 0.42, $\eta_{p}^{2}$ = 0.014) or error proportions (GD: 7.24% ± 4.49%, RGU: 6.56% ± 3.17%, NG: 7.54% ± 3.68%, *F*(2, 121) = 0.73, *p* = 0.49, $\eta_{p}^{2}$ = 0.012) among the GD, RGU, and NG groups. RT switch cost was the primary dependent variable, and ACC switch cost was used to assess possible speed–accuracy trade-offs.

Same as Experiment 1, the LMMs were fitted to participant-level condition means of RT switch costs rather than trial-level RT data. The primary linear mixed model examined the Group × RSI interaction and was specified as SwitchCost ~ Group × RSI + (1|ID). To assess the robustness of group-related effects, a second model further included weekly AVG hours and BDI score as grand-mean-centered covariates. Significant effects were followed by simple effects analyses, and post hoc comparisons were adjusted using the Holm correction method. Model diagnostics showed that all LMMs converged successfully without singular fits, with no serious deviations from residual normality or homoscedasticity.

### 3.2. Results

LMM omnibus tests revealed a significant main effect of RSI (*F*(3, 372) = 30.79, *p* < 0.001, $\eta_{p}^{2}$ = 0.199). Post hoc comparisons indicated that switch costs followed an overall downward trend as RSI increased; specifically, costs at 100 ms were significantly higher than at all other intervals, and costs at 3000 ms were significantly lower than at 600 and 1500 ms (*p*s < 0.01). Crucially, the Group × RSI interaction was significant (*F*(6, 372) = 2.47, *p* < 0.05,${\;\eta }_{p}^{2}$ = 0.038).

Decomposition of the two-way interaction (Figure 2b) revealed distinct temporal patterns across groups. For the GD group, switch costs decreased progressively as RSI lengthened from 100 to 3000 ms, reaching a negligible level at the longest interval (3.66 ms). In contrast, for the RGU and NG groups, the reduction in switch costs was confined to the 100–600 ms interval; further increases in RSI beyond 600 ms yielded no additional significant benefits.

Robustness analyses controlling for weekly AVG hours and BDI score yielded a similar overall pattern of findings (see Table 2), which supports H2. Notably, both covariates showed significant effects in the robustness models (*p*s < 0.05). To examine whether weekly AVG hours and BDI score were associated with the observed Group × RSI pattern, two additional LMMs were estimated: SwitchCost ~ Group × RSI × AVG + BDI + (1|ID) and SwitchCost ~ Group × RSI × BDI + AVG + (1|ID). When the interaction between Group and a covariate (i.e., AVG or BDI) was significant, follow-up probing analyses were conducted using the emmeans package. For continuous moderators, group differences were examined at low (−1 SD), mean, and high (+1 SD) levels of the moderator, and simple slopes within each group were estimated using emtrends, with Holm correction applied to multiple comparisons.

Results showed (see Table 3) that neither of the three-way interactions reached significance, suggesting that weekly AVG hours and BDI score did not significantly modulate the Group × RSI pattern. Nevertheless, significant Group × AVG, *F*(2, 124) = 4.47, *p* < 0.05, $\eta_{p}^{2}$ = 0.067, and Group × BDI, *F*(2, 124) = 3.17, *p* < 0.05, $\eta_{p}^{2}$ = 0.067, interactions were observed. Follow-up simple slope analyses indicated that weekly AVG hours was negatively related to switch cost in the NG group (*b* = −26.01, *p* < 0.01), but not in the GD (*b* = −1.05, *p* = 0.28) or RGU (*b* = −2.82, *p =* 0.10) group (Figure 2c). BDI was positively related to switch cost in the GD (*b* = 2.26, *p* < 0.05) and NG (*b* = 4.36, *p* < 0.05) groups, but not in the RGU (*b* = −1.75, *p* = 0.34) group (Figure 2d). Moreover, these findings were not explained by a speed–accuracy trade-off pattern (see Appendix A).

## 4. Discussion

The present study examined the cognitive control profile of AVG players with GD by dissociating proactive and reactive processes. Our findings supported H2 but did not support H1. Specifically, the results suggest that cognitive control in the GD group relied more heavily on a reactive, bottom-up late-correction mechanism, whereas proactive control, as a top-down early selection mechanism, was more fragile than in the RGU and NG groups. Specifically, when the interference resolution interval was extended, the GD group no longer benefited from prolonged cue-based preparation, unlike the stable proactive advantage observed in the other two groups. More notably, when this interval was extended to 3000 ms, switch cost in the GD group was reduced to a negligible level (i.e., 3.66 ms), whereas the RGU and NG groups still showed significant residual costs comparable to those at 600 ms. This near-zero switch cost may have more than one source. In addition to the dissipation of the previous task set, it may also reflect weaker stimulus-cued retrieval of competing task episodes in the GD group at longer intervals (Waszak et al., 2005). Taken together, these behavioral patterns suggest weakened proactive task-set maintenance and greater reliance on reactive control in GD players.

This reactive control bias is broadly consistent with dual process theory. According to this theory, addictive behaviors emerge when impulsive processes associated with stimulus-driven behavioral activation increasingly dominate over reflective processes involved in active goal maintenance (Strack & Deutsch, 2004; Hofmann et al., 2012; Zhou et al., 2022). At the cognitive-control level, this imbalance may manifest as weaker proactive control and greater reliance on reactive control. AVG experience may further reinforce this bias. Prior work has shown that AVG play decreases proactive control and induces changes in neural systems (G. L. West et al., 2017; R. West et al., 2020). AVGs are fast-paced and highly demanding; they may promote caudate-based learning, which supports increasingly stimulus–response–goals mappings, while reducing reliance on hippocampus-based learning, which is less favored under time pressure (Kühn & Gallinat, 2013; Vogel & Schwabe, 2016). As a result, AVG play may weaken sustained goal maintenance and increase reliance on late correction. Consistent with this view, prior research has shown that although AVG players can adapt control to task demands, they cannot maintain it proactively over a short delay (i.e., 2 s; Bailey et al., 2010).

Interestingly, our findings revealed that the cognitive advantage associated with AVG experience was evident only in the NG group, suggesting that such benefits may be more detectable at relatively low levels of AVG exposure than at higher levels. This is consistent with previous findings showing that the association between AVG experience and cognitive ability is not straightforward (Redick et al., 2017; Unsworth et al., 2015). A similar nonlinearity has also been observed for mental health outcomes: moderate gaming volume may be beneficial for mental well-being, but such benefits appear to diminish once daily gaming exceeds 3 h, and excessive playtime may even become a risk factor, with daily gaming time above 6 h being associated with higher anxiety symptoms (Egami et al., 2024; Chen et al., 2022). Taken together, gaming exposure benefits may vary across outcome domains and may not follow a simple linear pattern. Gaming hours may not provide a sufficiently sensitive index of gaming-related benefits, as AVG proficiency appears to predict performance better than AVG experience (Howard et al., 2024).

Additionally, our findings showed that higher levels of depression were associated with greater switch costs in the GD and NG groups, but not in the RGU group. A more plausible interpretation is that depressive symptoms generally weaken cognitive control, especially goal maintenance and task switching, thereby increasing switch costs (Snyder, 2013; Grahek et al., 2018). In the GD group, this association may reflect the joint effect of depression and greater reliance on reactive control. In the NG group, by contrast, this association may reflect a more general negative effect of depression on executive functioning. In the RGU group, the null association may be consistent with the possibility of emotion regulation through gaming, as gaming among RGU can enhance positive affect and reduce depression (Shin et al., 2012; Valadez & Ferguson, 2012), which may buffer the negative effect of depression on cognitive control.

This study has several limitations. First, our cross-sectional design cannot determine whether the reactive control bias is a result of AVG-related GD or a pre-existing risk factor. Future longitudinal studies are needed to establish causality. Second, Experiment 1 and Experiment 2 were completed in a fixed order within the same session, with a 10 min rest break between tasks. Although this procedure was identical across groups, possible practice or fatigue effects cannot be fully ruled out. Third, our sample consisted of young, non-clinical participants classified according to screening criteria, which limits the generalizability of these findings to clinical populations, other age groups, or other game genres. Fourth, because CSI and RCI/RSI manipulations provide behavioral indices rather than direct neural or physiological measures of proactive and reactive control, future research should combine such behavioral indices with neural or physiological data and broader assessments of executive, attentional, and affective functioning to clarify the mechanisms and generality of this control-mode shift.

Despite these limitations, our findings offer valuable practical implications. Interventions for AVG players with GD should specifically target proactive control, such as goal maintenance and anticipatory preparation, rather than just general executive functions. Furthermore, cognitive treatments should consider individual differences in gaming experience and depressive symptoms. This tailored approach can help distinguish healthy engagement from maladaptive gaming in action-based genres, ultimately leading to more effective prevention and intervention strategies.

## 5. Conclusions

In conclusion, AVG players with GD appear to rely more on reactive control and show relatively fragile proactive control, suggesting that impaired cognitive control in GD may reflect a shift in control mode rather than a global deficit.

## Figures and Tables

**Table 1:** Participant characteristics and between-group comparisons.

	**Experiment 1**					
	**GD (*n* = 33)**	**RGU (*n* = 38)**	**NG (*n* = 48)**	***F*/** $\chi^{2}$	$\eta_{p}^{2}$	**Post Hoc**
Age	19.76 (0.94)	20.00 (1.61)	20.19 (1.84)	0.74	0.013	-
Female: Male	22:11	24:14	27:21	0.97	0.090 ^a^	-
Education years	13.76 (1.00)	14.13 (1.56)	14.17 (1.84)	0.77	0.013	-
AVG hours	26.90 (14.56)	21.08 (7.83)	1.34 (1.30)	95.44 ***	0.622	GD > RGU > NG
non-AVG hours	4.62 (5.97)	3.99 (5.35)	0.87 (1.21)	8.74 ***	0.131	GD > NG, RGU > NG
DSM-5	6.27 (1.23)	2.37 (1.48)	1.50 (1.27)	134.25 ***	0.698	GD > RGU > NG
IAT	63.33 (8.14)	41.11 (6.94)	37.17 (9.10)	108.32 ***	0.651	GD > RGU > NG
BDI	17.09 (12.26)	9.32 (7.36)	8.67 (6.63)	10.38 ***	0.152	GD > RGU and NG
	**Experiment 2**					
	**GD (*n* = 36)**	**RGU (*n* = 38)**	**NG (*n* = 50)**	***F*/** $\chi^{2}$	$\eta_{p}^{2}$	**Post Hoc**
Age	19.72 (0.94)	20.03 (1.59)	20.08 (1.84)	0.61	0.010	-
Female: Male	24:12	24:14	27:23	1.57	0.112 ^a^	-
Education years	13.72 (0.97)	14.16 (1.55)	14.06 (1.83)	0.82	0.013	-
AVG hours	26.52 (14.22)	21.21 (7.75)	1.37 (1.31)	100.08 ***	0.623	GD > RGU > NG
non-AVG hours	4.32 (5.81)	4.02 (5.33)	0.74 (1.03)	9.30 ***	0.133	GD > NG, RGU > NG
DSM-5	6.25 (1.25)	2.42 (1.43)	1.40 (1.29)	148.15 ***	0.710	GD > RGU > NG
IAT	63.44 (7.93)	41.58 (6.32)	37.06 (9.01)	124.03 ***	0.672	GD > RGU > NG
BDI	16.22 (12.19)	9.50 (7.36)	8.54 (6.52)	8.92 ***	0.128	GD > RGU and NG

Standard deviation is shown in parentheses. IAT = Internet Addiction Test; BDI = Beck Depression Inventory. ^a^ Cramer’s V is reported as the effect size for the chi-square test of gender distribution. *** *p* < 0.001.

**Table 2:** Comparison results of main and robustness analysis.

	Model 1			Model 2		
Effect	*F*	*p*	$\eta_{p}^{2}$	*F*	*p*	$\eta_{p}^{2}$
Experiment 1:						
Group	1.51	0.23	0.025	1.03	0.36	0.017
CSI	100.13	<0.001	0.219	100.13	<0.001	0.219
RCI	18.66	<0.001	0.050	18.66	<0.001	0.050
Weekly AVG hours	-	-	-	0.18	0.67	0.002
BDI	-	-	-	0.12	0.73	0.001
Group × CSI	1.41	0.25	0.008	1.41	0.25	0.008
Group × RCI	0.31	0.73	0.002	0.31	0.73	0.002
CSI × RCI	6.11	0.01	0.017	6.11	0.01	0.017
Group × CSI × RCI	2.95	0.054	0.016	2.95	0.054	0.016
Experiment 2:						
Group	0.01	0.99	<0.001	0.81	0.15	0.013
RSI	30.80	<0.001	0.199	30.80	<0.001	0.199
Weekly AVG hours	-	-	-	4.09	0.045	0.032
BDI	-	-	-	4.94	0.03	0.038
Group × RSI	2.47	0.02	0.038	2.47	0.02	0.038

In Experiment 1, Model 1 = SwitchCost ~ Group × CSI × RCI + (1|ID), and Model 2 = SwitchCost ~ Group × CSI × RCI + AVG + BDI + (1|ID). In Experiment 2, Model 1 = SwitchCost ~ Group × RSI + (1|ID), and Model 2 = SwitchCost ~ Group × RSI + AVG + BDI + (1|ID).

**Table 3:** Results of the Three-Way Linear Mixed Models.

	Model 1			Model 2		
Effect	*F*	*p*	$\eta_{p}^{2}$	*F*	*p*	$\eta_{p}^{2}$
Group	4.91	<0.01	0.073	0.21	0.81	0.003
RSI	1.98	0.12	0.016	27.58	<0.001	0.182
Weekly AVG hours	11.52	<0.001	0.085	3.44	0.07	0.027
BDI	4.39	0.04	0.034	3.22	0.08	0.025
Group × RSI	1.53	0.17	0.024	2.26	0.04	0.035
Group × AVG	4.47	0.01	0.067	-	-	-
RSI × AVG	1.80	0.15	0.014	-	-	-
Group × RSI × AVG	1.25	0.28	0.020	-	-	-
Group × BDI	-	-	-	3.17	0.045	0.048
RSI × BDI	-	-	-	0.14	0.93	0.001
Group × RSI × BDI	-	-	-	0.21	0.97	0.003

Model 1 = SwitchCost ~ Group × RSI × AVG + BDI + (1|ID), and Model 2 = SwitchCost ~ Group × RSI × BDI + AVG + (1|ID).

## Data Availability

The data have been made available at https://osf.io/mwbas/overview?view_only=c06ebc4005ed4b24ad4326981d96edca (accessed on 11 June 2026).

## Appendix A. Supplementary Analyses of ACC Switch Costs

To complement the RT switch costs analyses reported in the main text, we conducted parallel analyses on ACC switch costs in Experiments 1 and 2 to examine whether the RT switch costs findings could be explained by a speed–accuracy trade-off. Specifically, a speed–accuracy trade-off would be suggested if the comparisons showing smaller RT switch costs in the main analyses also showed larger ACC switch costs in the corresponding ACC analyses. Therefore, in both experiments, we used the same analysis strategy as in the RT switch costs analyses. We first fitted a main linear mixed model and then fitted a robustness model by adding AVG and BDI as covariates. ACC switch costs were defined as the mean of two task-specific switch costs: color switch cost (color-repeat minus color-switch accuracy) and word switch cost (word-repeat minus word-switch accuracy). Larger values indicated larger ACC switch costs.

For ACC switch costs in Experiment 1. We fitted a main LMM with Group, CSI, and RCI as fixed effects: ACC switch costs ~ Group × CSI × RCI + (1|ID). The robustness model was specified as follows: ACC switch costs ~ Group × CSI × RCI + AVG + BDI + (1|ID).

The omnibus test of fixed effects in the main model showed a significant main effect of CSI, *F*(1, 357) = 18.27, *p* < 0.001,${\;\eta }_{p}^{2}$ = 0.049. ACC switch costs were lower at CSI 100 ms than at CSI 1000 ms (0.03 vs. 0.05). The Group × CSI interaction was also significant, *F*(2, 357) = 3.62, *p* < 0.05, $\eta_{p}^{2}$ = 0.02. The Group × CSI × RCI interaction was not significant, *F*(2, 357) = 0.56, *p* = 0.57,${\;\eta }_{p}^{2}$ = 0.003. Simple-effect analyses of the Group × CSI interaction showed that the interaction was mainly due to the reversed CSI effect in the NG group. Specifically, ACC switch costs were lower at CSI 100 ms than at CSI 1000 ms in the NG group (0.02 vs. 0.06, *t* = −4.97, *p* < 0.001, *d* = −0.73), whereas this difference was not significant in the IGD or RGU group (*p*s > 0.05). No significant group differences were found at either CSI level (*p*s > 0.05). The robustness model showed the same pattern of results (Table A1). Overall, the ACC switch cost results did not show a pattern opposite to the RT switch cost results reported in the main text. These findings therefore do not support the possibility that the RT switch costs findings in Experiment 1 were driven by a speed–accuracy trade-off.

For ACC switch costs in Experiment 2. We fitted a main LMM with Group and RSI as fixed effects: ACC switch cost ~ Group × RSI + (1|ID). We also fitted a robustness model as follows: ACC switch cost ~ Group × RSI + AVG + BDI + (1|ID).

The omnibus test of fixed effects in the main model showed a significant main effect of RSI, *F*(3, 372) = 16.24, *p* < 0.001,${\;\eta }_{p}^{2}$ = 0.116. Post hoc comparisons showed that ACC switch costs generally decreased as RSI increased, similar to the RT switch costs trend reported in the main text. Specifically, ACC switch costs were higher at 100 ms and 600 ms than at 1500 ms and 3000 ms (*p*s < 0.01), with no differences within each pair (100 vs. 600 ms; 1500 vs. 3000 ms; *p*s > 0.05). The Group × RSI interaction was not significant, *F*(6, 372) = 0.63, *p* = 0.70,${\;\eta }_{p}^{2}$ = 0.01. The robustness model showed the same pattern of results (Table A1). Overall, the ACC switch cost results did not show a pattern opposite to the RT switch cost results reported in the main text. These findings therefore do not support the possibility that the RT switch-cost findings in Experiment 2 were driven by a speed–accuracy trade-off.

**Table A1:** Comparison results of main and robustness analysis of switch costs in ACC.

	Model 1			Model 2		
Effect	*F*	*p*	$\eta_{p}^{2}$	*F*	*p*	$\eta_{p}^{2}$
Experiment 1:						
Group	0.10	0.90	0.002	0.04	0.96	0.001
CSI	18.27	<0.001	0.049	18.27	<0.001	0.049
RCI	0.86	0.35	0.002	0.86	0.35	0.002
Weekly AVG hours	-	-		0.01	0.94	<0.001
BDI	-	-		0.05	0.82	<0.001
Group × CSI	3.62	0.03	0.020	3.62	0.03	0.020
Group × RCI	0.87	0.42	0.005	0.87	0.42	0.005
CSI × RCI	1.12	0.29	0.003	1.12	0.29	0.003
Group × CSI × RCI	0.56	0.57	0.003	0.56	0.57	0.003
Experiment 2:						
Group	0.39	0.68	0.006	0.14	0.87	<0.001
RSI	16.24	<0.001	0.116	16.32	<0.001	0.090
Weekly AVG hours	-	-	-	0.22	0.64	<0.001
BDI	-	-	-	2.68	0.10	0.005
Group × RSI	0.63	0.70	0.010	0.63	0.70	0.008

In Experiment 1, Model 1 = SwitchCost ~ Group × CSI × RCI + (1|ID), and Model 2 = SwitchCost ~ Group × CSI × RCI + AVG + BDI + (1|ID). In Experiment 2, Model 1 = SwitchCost ~ Group × RSI + (1|ID), and Model 2 = SwitchCost ~ Group × RSI + AVG + BDI + (1|ID).
